# Impact of blended treatment literacy and psychoeducation on methadone maintenance treatment outcomes in Yunnan, China

**DOI:** 10.1186/s12954-016-0097-y

**Published:** 2016-02-26

**Authors:** Bo Zhang, Thomas Cai, Zhihua Yan, Gitau Mburu, Bangyuan Wang, Liping Yang

**Affiliations:** Yunnan Institute of Drug Abuse (YIDA), 300 Wujiadui, Xihuayuan, Xishan District, Kunming, Yunnan China; AIDS Care China, R 201, #17 Rongyuan, Jinjiang Residential Quarter, Bejing Road, Panlong District, Kunming, Yunnan China; International HIV/AIDS Alliance, 91-101 Davigdor Road, Hove, BN3 1RE UK; Department of Health Research, Lancaster University, Lancaster, LA1 4YW UK

**Keywords:** Methadone, Psychoeducation, Harm reduction, Injecting drug use, China

## Abstract

**Background:**

Outcomes of methadone maintenance treatment (MMT) in the management of opioid dependency can be impaired by poor adherence and retention, concomitant drug use, poor adjustment of methadone dosage, and low levels of awareness regarding methadone among drug users, among other factors. This study investigated the effects of intensive blended treatment literacy and psychoeducation on treatment compliance, methadone dose, and heroin use among MMT clients in China.

**Methods:**

A total of 492 MMT clients who tested positive for urine morphine at least once during a 12-week intervention period preceding the study were recruited from 16 MMT clinics. Employing a client-centred approach, a blended treatment literacy and psychoeducation intervention was then implemented between March and June 2014, comprising (1) intensified methadone treatment literacy sessions; (2) participatory goal setting; (3) continuous adherence monitoring and support; and (4) engagement of both peers and doctors in delivering psychoeducation. Wilcoxon signed-rank test was used to compare urine morphine positive rates, daily methadone dosage, and the number of days that clients successfully accessed methadone before and during the intervention.

**Results:**

During the intervention, urine morphine positive rates reduced to 27 % from 49.3 % previously; *p* < 0.001. In response to client needs, methadone dosages increased among 74 % of participants, remained unchanged among 12.0 %, and reduced among 13.4 % during the intervention. In addition, the average daily methadone dose increased from 63.0 to 72.6 mg; *p* < 0.001, while the average number of days that clients successfully accessed methadone increased from 69.4 to 73.9 over a period of 12 weeks; *p* < 0.001.

**Conclusions:**

Blended treatment literacy and psychoeducation delivered by a combination of peers and doctors was associated with reduced heroin use, improved treatment adherence, and higher methadone doses among our sample of MMT clients.

## Background

China has the largest population of drug users globally [1], and with it, rising levels of opioid dependency. In response, attempts to establish national coverage of opioid substitution therapy to treat opioid dependence gained momentum over the last decade. By 2012, there were nearly 750 methadone maintenance treatment (MMT) clinics in China, and more than 350,000 people had been provided with methadone since 2004 when the national programme was initiated [2].

Since methadone has similar physiological effects to opioids, it can help to reduce opioid dependence, overdose-related mortality, and opioid withdrawal symptoms [3]. However, despite it being used increasingly to tackle opioid dependency, its effectiveness depends on a variety of factors, including adherence and retention in MMT programmes [3, 4]. In addition, continued use of heroin and other drugs during MMT (which may itself result from inadequate methadone dosage) has been shown to have a negative impact on retention and long-term outcomes of MMT [5–7]. A recent review of MMT in mainland China showed that average retention rates between 2004 and 2012 were suboptimal, ranging from 30 % at 6 months in Shanghai to 70 % at 12 months in Xi’an [8]. These findings highlight the need for interventions to improve MMT adherence and retention.

There is an emerging consensus that optimising MMT outcomes requires a combination of effective behaviour change and structural and social interventions [8–10]. Regarding behavioural interventions, evidence suggests that psychosocial counselling, including cognitive behavioural therapy during MMT, can be beneficial for sustaining adherence and preventing relapse [9, 11]. However, there is some uncertainty regarding whether other more structured psychoeducation interventions can improve patient outcomes [12]. Psychoeducation is a client-centred approach of combining “empowerment of patients” with “active communication” of “scientifically founded treatment information” [13, 14]. Interest in treatment literacy is relevant given that a lack of awareness regarding MMT has been constantly associated with poor MMT results in China [8, 10, 15].

In addition, given the increasing focus on community-based methadone provision [16] and overdose prevention [17, 18], a consideration of the role that peers could play is timely. Peer-led outreach and support have proved to be useful in increasing uptake of methadone among hard-to-reach drug users [19], suggesting that peers could be beneficial in delivering structured psychoeducation interventions. This study explores the impact of blended treatment literacy and psychoeducation sessions provided by both doctors (fortnightly) and peers (weekly) on MMT outcomes: specifically, retention, concomitant heroin use during treatment, and methadone dosage.

## Methods

### Study setting

This study was conducted at 16 MMT clinics across Kunming city, Honghe, Dali, Chuxiong, Baoshan, and Dehong autonomous prefectures in Yunnan province. Intravenous heroin use is a major contributor to the spread of HIV in these six districts. The clinics were established between 2004 and 2008, and have cumulatively provided MMT to 11,928 people who use drugs, of whom 5647 currently are on methadone.

### Participant recruitment

Study participants were recruited from 16 MMT clinics across six districts in Yunnan province. Clinic clients were included if they (1) had received MMT in the study clinics for at least 12 weeks; (2) were aged over 18; (3) had tested for urine morphine at least twice and received a positive result at least once; (4) had understood the study information sheet and were willing to participate. Clients were excluded from the study if they would have been unable to receive the intervention due to detention.

### Intervention

The intervention consisted of blended treatment literacy and psychoeducation sessions provided to study participants fortnightly by doctors and weekly by peer educators between March and June 2014. The aim of the intervention was two-fold. First, it was to provide people who use drugs with knowledge and skills to manage their methadone treatment, injecting drug use, and related social and health issues. Second, it was to motivate participants to adhere to methadone treatment and, where possible, transition to a drug-free state.

Before the intervention was started, a total of 16 doctors and 32 peer educators from the 16 MMT clinics received a 2-day psychoeducation training provided by a psychiatrist. Overall, the intervention comprised (1) intensified treatment literacy sessions; (2) participatory goal setting; (3) continuous monitoring; and (4) engagement of peers and doctors in delivering psychoeducation. Goals were set through participatory discussions with clients and focused on adherence and achieving a drug-free state, while also motivating clients to take responsibility for their own health.

Following the training, doctors provided one-to-one and face-to-face treatment literacy on topics that included a general understanding of methadone and its effect on heroin addiction; differences between MMT and drug rehabilitation; appropriate methadone dosage; and common clinical problems during MMT and how to cope with them. Peer educators, some of who were former drug users, provided weekly intensified group psychoeducation sessions. Group sessions were held weekly at MMT clinics. Each MMT clinic had 2–3 active groups, each comprising 10–15 members. Group sessions covered treatment adherence, understanding of MMT, appropriate methadone dosage, and how to deal with heroin relapse. In addition, peers provided group psychoeducation sessions on the positive benefits of adherence to methadone; minimising withdrawal symptoms and craving for heroin; reducing or discontinuing heroin use; avoiding illegal and criminal behaviour; improving family and social relationships; re-establishing the ability to work; improving self-reliance and the ability to provide for families; and reducing the transmission of HIV through use of clean needles. Peer educators identified clients who were having treatment and other social difficulties based on: discussions during sessions, urine opioid-test results, and feedback from doctors. At the end of group sessions, peer educators engaged individual clients in setting goals related to adherence, drug use, and the above social and health issues. Through a client-centred approach, they discussed progress during follow-on one-to-one weekly sessions, and identified challenges and potential solutions based on each client’s circumstances. The intervention lasted for 12 consecutive weeks.

### Data collection

Data collected at the MMT clinics included urine morphine test results for a 12-week period before the intervention and the 12 weeks of the intervention. Urine morphine levels were tested once every 4 weeks before the intervention and every 2 weeks during the intervention. Urine samples were collected and tested in an assigned and private room at MMT clinics. Nurses conducted all the urine morphine tests using Morphine Gold Rapid Screen Test (colloidal gold). Additional data on the daily collection and average dose of methadone dispensed to participants was gathered from the Chinese National Methadone Maintenance Treatment Database.

### Data analysis

Data were analysed using SAS software, version 9.1. Descriptive summary statistics were obtained first. Because the distribution of the data was not normal, Wilcoxon signed-rank test was used to compare the following outcomes before and during the intervention: the urine morphine positive rate (skewness = 0.038, kurtosis = 0.757), the average number of days that methadone was collected (skewness = 0.995, kurtosis = 3.67), and the average daily dosage (skewness = 0.384, kurtosis = 7.796).

### Ethical considerations

When recruiting potential participants, study researchers gave MMT clients a study information sheet, read it aloud to them and explained potential benefits and risks, and invited them to participate. Researcher and clinic staff checked that clients understood that their participation would be voluntary. If a client was reluctant to participate, they were not persuaded. All clients were informed that their right to services at the clinics would remain unaffected regardless of whether they participated in the study or not. Participants were asked to provide urine samples as usual, but twice instead of once per month. Participants were not asked to provide other invasive biological samples. The study was approved by the Ethical Committee of Yunnan Provincial Methadone Maintenance Treatment Management Centre.

## Results

### Participant characteristics

A total of 492 people who use drugs participated in this study. The mean age was 39.8 years, 80.9 % were male, 48.6 % were either married or cohabiting, 50.2 % were educated to junior high school level, and 54.1 % were unemployed. The average duration of drug use before the study was 15.0 ± 7.1 years, while the average duration of MMT was 4.0 ± 2.4 years. A third of participants (33.5 %) had previously dropped out of MMT before they were recruited to this study (Table 1).

**Table 1:** Participants’ social-demographic characteristics

Socio-demographic characteristics		Number	Percentage (%)
Gender	Male	398	80.9
	Female	94	19.1
Age	20-29	47	9.6
	30-39	196	39.8
	40-49	197	40.0
	50 and above	52	10.6
Marital status	Unmarried	163	33.1
	Married or cohabiting	239	48.6
	Divorced	72	14.6
	Widowed	18	3.7
Educational level	Illiterate	16	3.3
	Primary school	139	28.3
	Junior high school	247	50.2
	Senior high school/technical school	78	15.8
	Junior college and above	12	2.4
Ethnicity	Han	369	75.0
	Hui	19	3.9
	Yi	13	2.6
	Other ethnicities	91	18.5
Occupation	Unemployed	266	54.0
	Farmer	146	29.7
	Self-employed	25	5.1
	Employed	19	3.9
	Other	36	7.3

### Concomitant drug use while receiving MMT

A total of 44.3 % of participants reported cigarette smoking or inhaling drugs; 43.3 % reported injecting drugs; and 12.4 % reported combining injecting drugs with either cigarette smoking or inhaling drugs.

### Provision and duration of the intervention

Between March and June 2014, a total of 8671 treatment literacy and psychoeducation sessions took place, 2929 of them delivered fortnightly by doctors and 5742 delivered weekly by peer educators. On average, each participant attended 17 treatment literacy and psychoeducation sessions, 5.96 times per capita from doctors and 11.67 from peer educators. Each one-to-one session delivered by a doctor lasted 15–20 min and each intensified session delivered by a peer educator lasted 30–60 min (Table 2).

**Table 2:** Duration of intervention

Clinic code	Sample size	Duration		Average sessions received	
		From	To	From doctor	From peer
1	27	03/04/2014	17/06/2014	5.6	11.48
2	46	03/04/2014	14/06/2014	6	12
3	25	02/04/2014	12/06/2014	5.88	11.64
4	29	27/03/2014	15/06/2014	6	12
5	31	06/04/2014	17/06/2014	6	12
6	27	06/04/2014	24/06/2014	6	12
7	28	06/04/2014	17/06/2014	5.93	11.93
8	26	06/04/2014	15/06/2014	5.85	11.58
9	27	04/04/2014	17/06/2014	5.96	11
10	37	18/03/2014	10/06/2014	6	11.92
11	27	27/03/2014	12/06/2014	6	11.82
12	35	26/03/2014	08/06/2014	6	12
13	37	31/03/2014	14/06/2014	6	12
14	39	31/03/2014	15/06/2014	5.95	10.98
15	29	03/04/2014	10/06/2014	6	12
16	22	16/03/2014	03/06/2014	6	9.73
Mean				5.96	11.67

### Intervention utilisation

More than 95 % of the 492 participants received all the possible treatment literacy and psychoeducation sessions during the 12-week intervention period (Table 3).

**Table 3:** Participant receiving intensified treatment literacy and psychoeducation

Provider category	Number of sessions	Number of participants	Percentage (%)
From doctors	3 sessions	1	0.2
	4 sessions	5	1.0
	5 sessions	10	2.0
	6 sessions	476	96.8
	Total	492	100.0
From peer educators	<5 sessions	1	0.2
	5–10 sessions	16	3.3
	11–12 sessions	475	96.5
	Total	492	100.0

### Changes in treatment outcomes before and during the intervention

We compared the urine morphine positive rate (the positive person-times of all urine morphine test person-times), the number of days that participants received methadone, and daily methadone dosages before and during the 12-week intervention (Table 4).

**Table 4:** Changes in treatment outcomes among participants before and during the intervention

Treatment outcome	Before intervention	During intervention	*S*	*p* value
Urine morphine positive rate (%)	49.3 (694/1408)	27.0 (784/2905)	35,231.5	<0.001
Daily dosage of methadone (mg)	63.0	72.6	−32,123	<0.001
Average number of days methadone accessed (per client)	69.4	73.9	−19,000	<0.001

### Opioid-using behaviour

Urine morphine test results can disclose the opioid-using behaviour of participants. While all participants (100.0 %) had tested positive urine morphine at least once during the 12 weeks before the intervention, only 66.7 % (*n* = 328) tested positive during the three months of the intervention. The frequency of positive urine morphine tests decreased among 362 participants (73.6 %), remained unchanged among 63 participants (12.8 %), and increased among 67 (13.6 %) participants. Before the intervention, a total of 1408 urine morphine tests were conducted, with 694 testing positive, while during the intervention there were 2905 tests, with 784 being positive. Thus, urine morphine positive rate decreased from 49.3 % (694/1408) to 27 % (784/2905). A Wilcoxon signed-rank test comparing urine morphine positive rates before and during the intervention resulted in *S* = −35,231.5, *p* < 0.001, showing that urine morphine positive rate was significantly lower during the intervention as compared to before, at a two-sided alpha of 0.05.

### Changes in daily methadone dosage

A comparison of the daily methadone dosage of participants before and during the intervention was conducted. Of the total 492 participants, 367 (74.6 %) had their daily methadone dosages increased, 59 (12.0 %) remained unchanged, and 66 (13.4 %) had their doses reduced. The average daily methadone dosage was 63.0 ± 28.0 mg before and 72.6 ± 32.9 mg during the intervention, showing a 9.6 mg increase. A Wilcoxon matched-pairs signed-rank test comparing methadone dosage before and during the intervention resulted in *S* = −32,123, *p* < 0.001. In conclusion, the daily methadone dosage was significantly higher during the intervention compared to the period before, at a two-sided alpha level of 0.05.

### Number of days receiving methadone treatment

We compared the number of days that participants took methadone treatment over the 12-week period before the intervention and during the 12 weeks of the intervention. This number increased among 267 (54.3 %) participants, remained unchanged among 94 (19.1 %) and decreased among 131 (26.6 %) participants. The average number of days that participants took MMT was 69.4 ± 16.9 during the 12 weeks before the intervention, and increased to 73.9 ± 13.5 during the intervention: an increase of 4.5 ± 12.7 days. A Wilcoxon signed-rank test comparing the number of days that participants received methadone before and during the intervention resulted in *S* = −19,000, *p* < 0.001. Thus at a two-sided alpha level of 0.05, the number of days that participants received methadone was significantly higher during the intervention compared to before the intervention.

## Discussion

Findings from this study suggest that a blended treatment literacy and psychoeducation intervention that involves both trained healthcare providers and peers can improve MMT outcomes. In this study, doctors and peer educators provided intensified treatment literacy and psychoeducation sessions to MMT clients with a history of relapse, covering both clinical and social aspects of MMT. The results indicated that this intervention reduced their tendency to relapse, increased their methadone dosage, and improved their treatment compliance.

Although MMT is effective for opioid dependence [20], identifying an optimal dosage is an important determinant of its outcomes [21, 22]. In our study, MMT clients were educated on how adjusting their methadone dose could help them to cope with withdrawal symptoms, and were involved in decisions related to graduating their doses. This approach may have reduced the tendency of MMT clients to relapse. Research from other settings suggests that a flexible dosing strategy, allowing for methadone dose adjustment according to clients’ needs, is often associated with better retention outcomes [23, 24].

Previous studies have shown that counselling interventions are necessary but insufficient on their own for reducing heroin use and other risk behaviours [25]. They have also been shown to be less effective on their own compared to MMT [26]. While some scholars have stressed the need for integrating counselling into MMT [27, 28], others have explored the impact of extending the duration of such counselling to improve adherence to both MMT and antiretroviral drugs [29]. In our study, psychoeducation was combined with treatment literacy, with an emphasis on intensive communication of scientifically sound treatment information and goal oriented psychoeducation, monitoring and support. The engagement of peers, some of who were former drug users, to provide the intervention may have been particularly motivating and relevant to clients, while allowing a client-centred approach of identifying challenges and solutions to be implemented.

All participants in this study were recruited on the basis of testing positive at least once during routine urine morphine tests over the 12 weeks period prior to the intervention. During the 12-week intervention, a third of participants (33.3 %) did not test positive for urine morphine at all, and overall, urine morphine positive rate decreased from 49.3 to 27 %. At the same time, the daily methadone dosage and treatment compliance increased, validating the observed decrease in the urine morphine positive rate. Our findings are supported by other studies in the Chinese context showing that psychological interventions, such as cognitive behavioural therapy and supportive mental health care can reduce the incidence of relapse and improve the quality of life of people who use heroin [9, 30, 31]. What is unique to our study is that unlike other contexts where nurses and doctors provide these interventions, our intervention utilised peers to provide some sessions, with good results.

However, it should be noted that during the intervention period, the urine morphine positive rate increased among a minority (13.6 %) of participants; 13.4 % of participants took a reduced daily methadone dosage; and 26.6 % of participants accessed methadone on fewer number of days than they did prior to the intervention. This perhaps reflects the existence of other factors that influenced compliance with MMT, despite provision of intensive blended treatment literacy and psychoeducation sessions. Other studies have shown, for instance, that structural factors related to the nature of MMT clinics themselves may have an effect on relapse [21, 32]. Therefore, further research is required among clients with poor outcomes to explore the reasons for these and to identify tailored interventions for them.

### Limitations

While the urine morphine test result was a proxy indicator of intervention outcomes in terms of reducing relapse into injecting drug use, it is possible that participants could predict their scheduled urine test and reduce their tendency to use heroin during these periods, thereby producing a spurious intervention effect. The lack of a comparison arm limits the extent to which our assertions of impact can be made. In addition, the observational study design may not have eliminated other confounding factors that a randomised control study would have achieved.

## Conclusions

In this study, blended treatment literacy and psychoeducation delivered by a combination of peers and doctors was associated with reduced heroin use, improved treatment compliance, and increased methadone dosage among our sample of MMT clients. We hope that further studies exploring treatment literacy and psychoeducation in combination with social structural interventions will be conducted among other MMT clinics in China, so that the evidence base is strengthened and MMT outcomes further improved.
